# Calcium Enabled Remote Loading of a Weak Acid Into pH-sensitive Liposomes and Augmented Cytosolic Delivery to Cancer Cells via the Proton Sponge Effect

**DOI:** 10.1007/s11095-022-03206-0

**Published:** 2022-02-28

**Authors:** Mimi M. Yang, Sasi Bhushan Yarragudi, Stephen M. F. Jamieson, Mingtan Tang, William R. Wilson, Zimei Wu

**Affiliations:** 1 School of Pharmacy, The University of Auckland, Auckland, 1142 New Zealand; 2 Auckland Cancer Society Research Centre, The University of Auckland, Auckland, New Zealand

**Keywords:** Calcium acetate, Cytosolic delivery, Co-localization analysis, Endosomal entrapment, pH-sensitive liposomes, Proton sponge effect, Remote drug loading

## Abstract

While delivery of chemotherapeutics to cancer cells by nanomedicines can improve therapeutic outcomes, many fail due to the low drug loading (DL), poor cellular uptake and endosomal entrapment. This study investigated the potential to overcome these limitations using pH-sensitive liposomes (PSL) empowered by the use of calcium acetate. An acidic dinitrobenzamide mustard prodrug SN25860 was used as a model drug, with non pH-sensitive liposomes (NPSL) as a reference. Calcium acetate as a remote loading agent allowed to engineer PSL- and NPSL-SN25860 with DL of > 31.1% (w/w). The IC_50_ of PSL-SN25860 was 21- and 141-fold lower than NPSL and free drug, respectively. At 48 h following injection of PSL-SN25860, NPSL-SN25860 and the free drug, drug concentrations in EMT6-*nfsB* murine breast tumors were 56.3 µg/g, 6.76 µg/g and undetectable (< 0.015 µg/g), respectively (n = 3). Meanwhile, the *ex vivo* tumor clonogenic assay showed 9.1%, 19.4% and 42.7% cell survival in the respective tumors. Live-cell imaging and co-localization analysis suggested endosomal escape was accomplished by destabilization of PSL followed by release of Ca^2+^ in endosomes allowing induction of a proton sponge effect. Subsequent endosomal rupture was observed approximately 30 min following endocytosis of PSL containing Ca^2+^. Additionally, calcium in liposomes promoted internalization of both PSL and NPSL. Taken together, this study demonstrated multifaceted functions of calcium acetate in promoting drug loading into liposomes, cellular uptake, and endosomal escape of PSL for efficient cytoplasmic drug delivery. The results shed light on designing nano-platforms for cytoplasmic delivery of various therapeutics.

**Graphical abstract:**

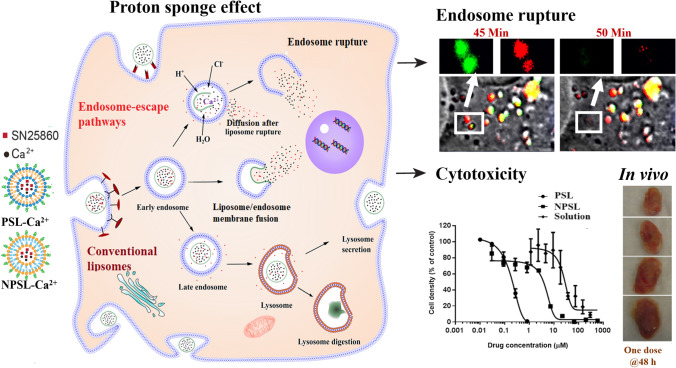

## Introduction

Cytoplasmic delivery of chemotherapeutic agents to cancer cells using nanoparticles can maximize therapeutic efficacy and safety [1]. However, despite substantial advances and considerable research efforts in cancer nanomedicine [2, 3], the successful translation rate is low^4^. Most cancer nanomedicines that have been approved have an improved safety profile and patient quality of life but have marginal improved efficacy compared to standard therapies [4]. Current cancer nanomedicines frequently fail to deliver sufficient chemotherapeutics to their target inside the cells, primarily due to 1) inefficient cellular uptake, 2) entrapment and digestion in the endosomal-lysosomal system following endocytosis, and 3) low drug loading (usually less than 10% w/w) [5, 6].

Endocytosis is a natural phenomenon for cellular uptake of nanoparticles by membrane invaginations, leading to the formation of endosomes [7, 8]. Mature endosomes acidify and fuse with lysosomes, leading to sequestration and degradation of the nanomedicine by lysosomal enzymes, a process known as endosomal entrapment [1, 9]. Thus, efficient nanomedicines that have not only high drug loading [10], but also the ability to enter cancer cells followed by rapid release of the payload to circumvent “endosomal escape” are highly sought to enable effective delivery of chemotherapeutics to their targets [9, 11].

In recent years, pH-sensitive liposomes (PSL) have been extensively investigated to address the endosomal entrapment of conventional liposomes [12, 13]. PSL leverages the progressive acidification of endosomes (pH 5–6.5) and rapidly releases payloads in acidic environments [14, 15]. PSL consisting of 1,2-dioleoyl-sn-glycero-3-phosphoethanolamine (DOPE) and cholesteryl hemisuccinate (CHEMS) are preferred due to their preferential uptake into tumor cells, attributed to the fusogenic property of DOPE [16, 17]. In general, pH-sensitive nanoparticles promote endosomal escape by three hypothetical pathways: i) liposomal destabilization in the acidic lumen of endosomes, ii) fusion of liposomes with endosomal membranes, and iii) rupture of endosomal membranes [18]. Our previous studies demonstrated that the DOPE-CHEMS-based PSL enter cells mainly through clathrin-mediated endocytosis [19], followed by endosomal escape primarily by destabilization in the acidic lumen and fusion with the endosomal membrane [19, 20]. DOPE may also destabilize endosomal membranes via its protonated head group [21]. However, even endowed with pH-sensitivity, as well as fusogenic and membrane destabilizing properties, it seems only a small fraction of PSL are capable of crossing the endosomal membrane, making endosomal entrapment still a bottle-neck for the PSL mediated cytosolic delivery of chemotherapeutics [20, 22, 23].

Calcium acetate is commonly used for remote loading of weakly acidic drugs into liposomes by establishing a transmembrane gradient and promoting an influx of drug moecules [24, 25]. With this method we have previously loaded an acidic drug candidate SN25860 with a p*K*a of 4.1 into a DOPE-CHEMS based PSL and non pH-sensitive liposomes (NPSL). SN25860 is a dinitrobenzamide mustard prodrug which is activated via bioreduction to form DNA-crosslinking metabolites [26]. The antiproliferative potency against EMT6-*nfsB* breast cancer cells of the PSL formulation of SN25860, with DL of 7% w/w, was found to be 21- and 24 times higher than that of the NPSL and the free drug solution, respectively [19].

From a physiological perspective, endocytosed calcium ions (Ca^2+^) in the endosomal compartments can cause the entry of protons (H^+^) from the cytosol when Ca^2+^ exit via the endosomal calcium channels. This is followed by an influx of chloride ions (Cl^−^) (driven by an electrical potential difference) accompanied by water, which induces a high osmotic pressure and eventually leads to endosomal rupture, known as the proton sponge effect [27]. The principle has been used to explain the high efficiency for endosomal escape of DNA and siRNA from nanoparticles or nanocomplexes containing calcium salts (phosphate or carbonate) or calcium ions [28–30]. However, complete disruption of the endosomal membrane as proposed by the proton sponge effect with lipid/liposomal nanoparticles is under debate mainly due to a lack of supporting evidence [31–35]. Hence, an alternative endosomal escape mechanism for lipid based formulations that relies on lipid-membrane interactions and formation of transient and local perturbations of the endosomal membrane has been proposed [36, 37]. However, again evidence is elusive. Therefore, to improve the design of nanomedicines it is important to fully reveal the mechanisms involved in endosomal escape.

In the present study, PSL-SN25860 with high DL were developed using calcium acetate as remote loading agent by modification of a previously reported method^19^ with additional drug loading strategies [10, 38]. NPSL-SN25860 with similar DL, and a cyclodextrin-enabled drug solution formulation (free drug) were developed as reference formulations. EMT6-*nfsB* breast cancer cells were used for *in vitro* cytotoxicity studies and for the establishment of tumor models for anti-tumor efficacy and tumor biodistribution studies. The cell line, stably transfected to express *E. coli* nitroreductase *nfB,* has been used to evaluate the anti-tumor activities of dinitrobenzamide mustard prodrugs [26]. To develop insight into how calcium promotes endosomal escape and thus enhances cytosolic delivery in tumor cells, live-cell imaging of EMT6-*nfsB* cells was employed to visualize the intracellular trafficking particularly the endosomal escape pathway of PSL in comparison with NPSL with the same payloads.

## Materials and Methods

### Materials

SN25860, 3-(5-(bis(2-bromoethyl)amino)-2,4-dinitrobenzamido)propanoic acid, was designed and synthesized at Auckland Cancer Society Research Centre (ACSRC), University of Auckland. DOPE, CHEMS 1,2-distearoyl-sn-glycero-3-phosphocholine (DSPC), 1,2-dipalmitoyl-sn-glycero-3-phosphocholine (DPPC), N-(carbonyl-methoxy-polyethylene-glycol-2000)-1,2-distearoyl-sn-glycero-3-phosphoethanolamine (DSPE-mPEG_2000_) and 1,2-dioleoyl-sn-glycero-3-phosphoethanolamine-N-(lissamine rhodamine B sulfonyl) (Rh-PE, ammonium salt) were purchased from Avanti Polar Lipids (Alabama, USA) while cholesterol (CHOL) from Sigma-Aldrich Ltd (Auckland, New Zealand). Hydroxypropyl-β-cyclodextrin (HP-β-CD) was a gift from Shandong Binzhou Zhiyuan Biotechnology Co., Ltd, China. Milli-Q water was prepared using a water purification system (Millipore Corp., MA, USA).

Mouse mammary carcinoma cells (EMT6-*nfsB*) were from ACSRC. For cell staining, Hoechst 33,342, and LysoTracker Deep Red (short for LysoTracker) were purchased from Thermo Fisher Scientific (MA, USA). All other reagents were of analytical grade except acetonitrile which is of chromatographic grade.

All animal studies were performed under approval from the University of Auckland Ethics Committee (Ethics Approval Number 001593) at the animal study facility, Vernon Jenson Unit (VJU) of the University.

### Liposome preparation and remote drug loading

The blank PSL composed of DOPE: CHEMS: DSPC: CHOL: DSPE-mPEG2000 (molar ratios 4:2:2:2:0.3) and NPSL composed of DPPC: CHOL: DSPE-mPEG2000 (molar ratios 6:4:0.3) were prepared using the thin film hydration method^10,19^ with modification. The lipid film was hydrated with a calcium acetate solution (250 mM or 500 mM, pH adjusted to 9) for 45 min at 45 °C. The coarse liposome suspension was then subjected to seven freeze (2 min)-and-thaw (7 min) cycles and extruded through double stacked 0.1 and 0.08 µm membranes (10 times each) using a 10 mL LIPEX™ Extruder (Northern Lipids Inc., Canada). The resulting liposomes had free acetate removed by dialysis and were subsequently used for drug loading.

Blank PSL and NPSL were prepared by following same steps but were hydrated with phosphate buffer (0.1 M, pH 7.4, isotonic). For intracellular trafficking studies, liposomes were labelled with Rh-PE (MW 1301.7) at 0.5 µg/10 mg liposomes in the bilayer by adding Rh-PE during thin film preparation.

To optimize DL into liposomes, the effects of drug loading time and temperature, 40 -60 °C were compared. SN25860 has a poorly water solubility (50 µg/mL). We have previously achieved a DL of 7%w/w by increasing the solubility with the aid of cyclodextrins to 0.5 mg/ml in the loading medium^19^. To further increase DL, in this study, concentration of SN25860 was enhanced up to 4.5 mg/ml by combining 2–3% HP-ß-CD and 2% ethanol alongside pH-adjustment with phosphate buffer (pH 7.0).

After drug loading the liposomes were subject to ultracentrifugation at 188,272 × *g*, 4 °C for 1 h (WX Ultra 80, Thermo Fisher Scientific, USA). The drug loaded liposomal pellets of PSL and NPSL were obtained and stored in pellet form or suspended in PBS at 4 °C in the dark before further use.

### Physicochemical characterization of liposomes

Particle size, polydispersity index (PDI), and zeta potential of liposomes were measured using Malvern Nano ZS (Malvern Instruments, UK) after appropriate dilution with water. All measurements were conducted in triplicates at 25 °C. The morphology of blank and high drug loaded liposomes were analyzed by cryogenic transmission electron microscopy (cryo-TEM). Samples were visualized on a Tecnai 12 electron microscope (FEI, Hillsboro, USA) operating at 120 kV. Observations were made of the bilayered membrane as well as drug precipitation in the liposome core.

To determine entrapment efficiency (EE) and DL, liposome pellets were dissolved in PBS with 10% Triton X-100 followed by sonication for 15 min. The amount of SN25860 in liposomes was obtained by a validated stability-indicating high performance liquid chromatography (HPLC) method^19^. The EE and DL were calculated as:

$$\begin{array}{c}\mathrm{EE }\left(\mathrm{\%}\right)=\frac{\mathrm{mass of drug in liposomes}}{\mathrm{mass of drug used for loading}}\times 100\\ \mathrm{DL }\left(\mathrm{\%}\right)=\frac{\mathrm{mass of drug in liposomes}}{\mathrm{mass of drug loaded liposomes}}\times 100\end{array}$$

### pH-responsiveness

The drug release profiles of SN25860 loaded into PSL and NPSL were determined using the dialysis method. The liposomes (equivalent to 10 mg lipids) were dispersed in 1 mL of PBS (50 mM, pH 7.4) and sealed in a cellulose acetate dialysis tubing (MWCO 12–14 kDa). Then the dialysis tubes were immersed in 50 mL of release medium (PBS 50 mM of pH 7.4, 6.5 and 5.5) and incubated at 37 °C with shaking at 100 rpm for 48 h. Aliquots (100 µL) of release media were withdrawn at pre-determined intervals and replaced with the same volume of fresh medium. The concentration of released SN25860 was determined by HPLC^19^.

### *In vitro* cytotoxicity

Cytotoxicity of PSL and NPSL with or without drug loaded in mouse mammary carcinoma cells (EMT6-*nfsB*) was evaluated by sulforhodamine B (SRB) assay. The *nfsB*-transfected mouse mammary carcinoma cell line ^26^ were cultured in αMEM, supplemented with 5% FCS. The cells were confirmed to be mycoplasma-free by PCR-ELISA (Roche Diagnostics).

Briefly, 150 cells in 100 µL aliquots (determined by a Beckman Coulter) were plated in 96-well microtiter plates. Following 24 h culture, during which cells attached and resumed growth, 100 µL of the tested formulations (drug solution, NPSL or PSL) re-suspended in PBS (pH 7.4) at different ratios before further diluted with culture medium and added to each well (*n* = 3). The cells were treated for 18 h before drug was washed out with culture media followed by SRB assay^19^. Cells treated with culture medium was used as negative controls. The IC_50_ value of each formulation was determined using GraphPad Prism 8.0.0 (GraphPad Software Inc., U.S.A). The experiment was triplicated.

### Safety, tumor distribution and anti-tumor efficacy in tumor-bearing mice

To pilot the systemic toxicity, liposomal formulations were intravenously administered via the tail vein to BALB/c mice (*n* = 3) and, as tumors did not grow, then CD-1 mice (*n* = 3) at 1 mmol/kg of drug (40 mg phospholipids). An injectable solution of SN25860 (4 mg/ml) was obtained using combination solubilization approach, 2.5% HP-β-CD, 2% ethanol with pH adjusted to 7.0 with phosphate buffer. The animals were observed for up to 2 weeks for morbidity and mortality.

Female CD-1 nude mice were inoculated subcutaneously with 5 × 10^6^ EMT6-*nfsB* cells. The length (L) and width (W) of tumors were used to estimate the volume of tumor (V) $\left(\mathrm{V}=\mathrm{L}\times {\mathrm{W}}^{2}\times 0.52\right)$. Once tumors grew to approximately 200 mm^3^, mice were randomized to treatment groups, NPSL, PSL and free drug; PBS as control (*n* = 3); mice were dosed with formulations at the predetermined dose of 1 mmol/kg (40 mg phospholipids) via tail vein (100 µl/10 g of body weight). After 48 h, mice were sacrificed, tumors and major organs were excised for drug analysis. Briefly, each of the organs was added to acetonitrile (0.2 g/mL) and homogenized by a tissue dissociator (gentle MACS Dissociator, Miltenyi Biotech) at 2,000 × *g* for 1.5 min; samples were then centrifuged at 10,000 × *g*. The supernatant was dried and residual dissolved in 50 µl mobile phase to determine the SN25860 concentration by HPLC. A standard curve prepared with a concentration range 0.05—5 µg/ml (0.1—10 µM), spiked with mouse tissue samples. The limit of detection for the assay was found to be 15 ng/mg in tissues.

For the anti-tumor efficacy study, tumor bearing mice were randomized to treatment groups (6–7 mice each group). Free SN25860 solution, NPSL, PSL were intravenously injected via tail vein at 1 mmol/kg (512 mg/kg). A control group (*n* = 3 mice) were dosed with PBS at the same volume. After 48 h, mice were sacrificed, anti-tumor efficacy was evaluated by an ex-vivo clonogenic assay^26^. Experimentally, tumors were dissected under aseptic conditions by submerging the animal body briefly in 80% ethanol and removing the tumors in a sterile hood. The excised tumors were minced before dissociation with an enzyme cocktail (Pronase 2.5 mg/ml, Collagenase 1 mg/ml, DNAase 0.2 mg/ml) and incubated at 37 °C for 45 min. After tumor dissociation, cells were collected by low-speed centrifugation and cell density was determined by hemocytometer or electronic particle counter. A known number of cells (1 × 10^5^ cells followed by a sixfold dilution down to 460 cells) were plated onto P-60 tissue culture plates containing α-MEM + 5% FCS + 3 µM puromycin and grown for 7 days in 5% CO_2_ incubator at 37 °C to assess clonogenic survival. After incubation, cells were stained with 2% methylene blue in 50% ethanol. Dilutions providing between 10 and 100 colonies/dish were counted and plating efficiency (PE, the % of cells seeded into each dish that grows to form a colony) was calculated as the ratio of the number of colonies to the number of cells inoculated.

### Live cell imaging of EMT6-*nfsB* cells treated with liposome formulations

To investigate the effect of calcium loaded liposomes on endosomal escape, live cell imaging was performed using the Olympus Fluroview FV1000 CLSM. EMT6-*nfsB* cells were seeded in ibidi 8-well chambered slides at a density of 10^3^/well in 300 µL medium, and cultured for 48 h. The cells were stained by LysoTracker (100 nM) for 90 min and Hoechst 33,342 (1 mg/mL) for 20 min, both at 37 °C. Cells with a clear morphology were chosen in a differential interference contrast (DIC) channel. Cells were treated with Rh-PE labelled liposomes at a total lipid concentration of 50 µg/mL and immediately observed using CLSM with a 63 X oil immersion objective and live cell incubator system. Untreated cells stained with LysoTracker (100 nM) served as control. Confocal images were acquired at regular intervals of 15 min over a 2 h period. Images were analyzed using Zen Blue software (Carl Zeiss, Germany). The intensity of LysoTracker, Rh-PE and their co-localization was quantified using ImageJ software, version 1.6 (Bethesda, Maryland, USA) ^22^. The co-localization threshold plugin was used to calculate Pearson’s correlation coefficients (*r*) and generate a scatterplot of overlapping intensities where the darker pixels represent less frequent occurrences of pixel intensity while yellow indicates a higher frequency of co-localization. Graphs for *r* over time lapse were plotted by importing the tabular results from ImageJ into GraphPad Prism for data analysis for formulation effects.

### Statistical analysis

One-way analysis of variance (ANOVA) with Tukey’s multiple comparison test was performed to determine the difference between groups using GraphPad Prism 8.0.0 (GraphPad Software Inc., La Jolla, USA). The level of significance for all statistical analysis was set at 0.05.

## Results

### Characterization of liposome formulations

Both high EE and DL were achieved (Table 1) by further optimization of previous drug loading conditions^19^, including increasing the concentration of calcium acetate to 500 mM, and of SN25860 in the loading medium to 4.5 mg/ml with addition of 2.5% (w/v) HP-β-CD and 2% ethanol with pH maintained at 7.0 using PBS. The temperature of drug loading medium was 55 °C for PSL and 47 °C for NPSL and the loading time was 60 min for both PSL and NPSL. This led to the highest DL (w/w) of > 30% (Table 1). Reducing HP-β-CD concentration to 2% decreased DL to 25.4 ± 2.8% while a further increase of HP-β-CD to > 3% reduced liposome pellet size, as it can disrupt the integrity of liposomal bilayer[39]. Decreasing the calcium acetate concentration to 250 mM reduced DL to 13% for both liposomes, while without the buffer the DL was 21.9 ± 2.4% and 24.6 ± 3.1 for PSL and NPSL respectively.

**Table 1:** Physical characterization of PSL and NPSL containing PBS (pH 9) or 500 mM calcium acetate (Ca) with or without SN28560 loading (n = 3–6). (NB: reducing Ca to 250 mM did not change the property of PSL-Ca/NPSL-Ca)

Formulation	Size (nm)	PDI	Zeta Potential (mV)	EE (%)	DL (%, w/w)
PSL-PBS	131.7 ± 2.7	0.09 ± 0.03	2.17 ± 1.2	-	-
PSL-Ca	138.7 ± 5.7	0.09 ± 0.04	4.79 ± 3.1	-	-
PSL-SN28560*	168.9 ± 4.2	0.20 ± 0.02	0.04 ± 0.01	85.9 ± 1.4	31.1 ± 1.3
NPSL-PBS	131.4 ± 1.8	0.04 ± 0.01	3.27 ± 2.7	-	-
NPSL-Ca	133.2 ± 3.2	0.08 ± 0.02	5.27 ± 2.1	-	-
NPSL-SN28560*	165.5 ± 5.1	0.15 ± 0.04	0.08 ± 0.03	95.1 ± 0.4	± 1.4

*PSL-SN28560 and NPSL-SN28560 were formulated with 500 mM calcium acetate as remote loading agent. “-”: not applicable.

**Fig. 1:**
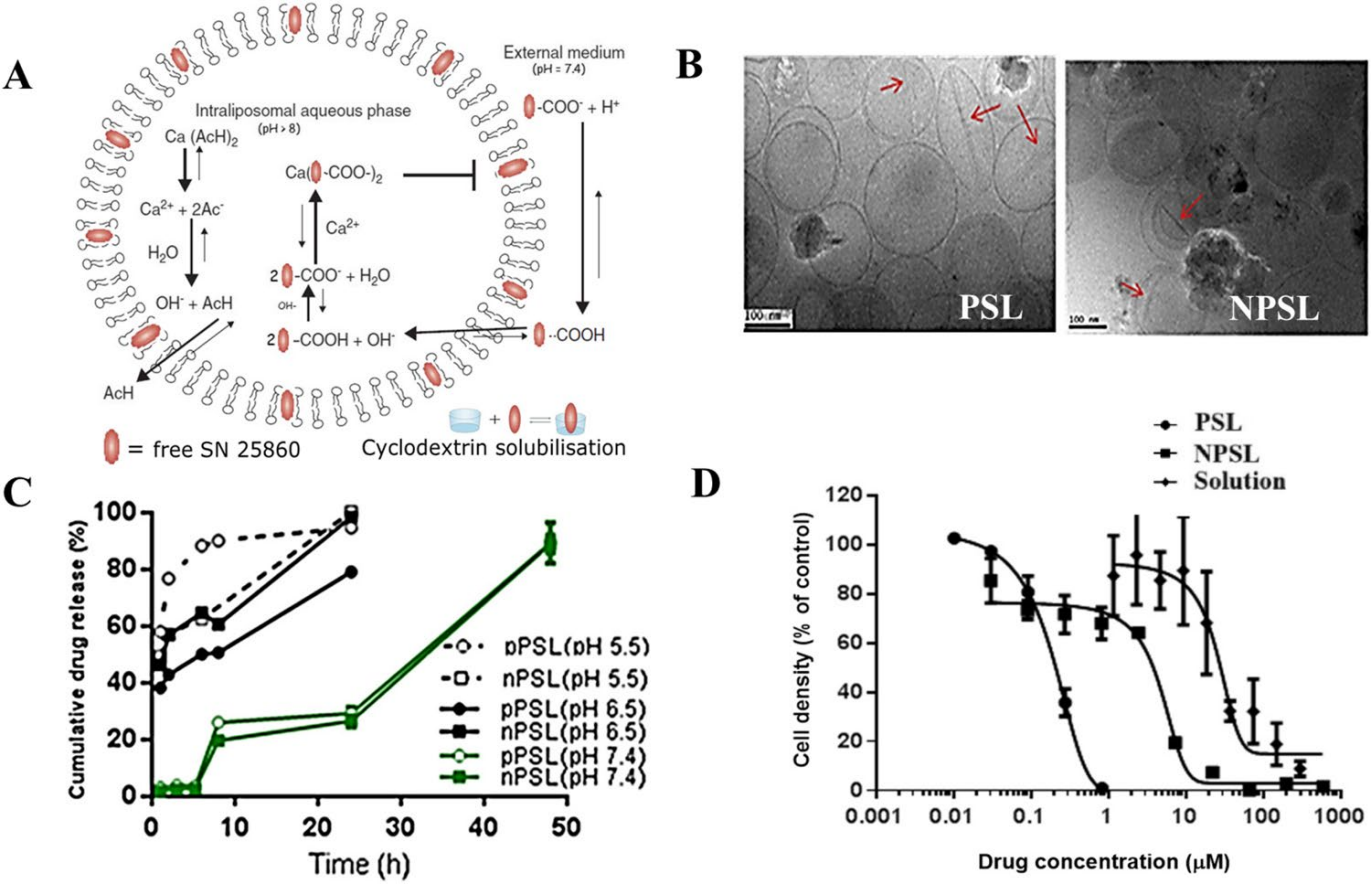
Physicochemical characterization and cytotoxicity to EMT6-*nfsB* breast cancer cells of PSL-SN25860 and NPSL-SN25860 using calcium acetate as a remote loading agent. A) Mechanism for drug loading. B) Representative cryo-TEM micrographs. The red arrows indicate drug precipitates or ‘bundles’ in the liposome cores. C) *In vitro* pH responsive drug release profiles (mean ± SD; *n* = 3); and D) Growth curves of EMT6-*nfsB* cells treated with free drug, PSL and NPSL for 18 h (means ± SEM; *n* = 3 individual experiments). Blank liposomes were non-toxic at the corresponding concentration range

The size and zeta potentials of PSL and NPSL containing 500 mM calcium acetate (PSL-Ca and NPSL-Ca) measured by dynamic light scattering (DLS) were shown in Table 1. The particle size significantly increased after remote drug loading (Fig. 1A). Cryo-TEM images of drug loaded PSL and NPSL showed that the liposomes were unilamellar and generally uniform in size with drug precipitate inside the liposome cores (indicated by red arrows in Fig. 1B).

PSL demonstrated a pH-dependent release profile (Fig. 1C). At pH 7.4, 70% of drug remains entrapped in the first 24 h. At endo-lysosomal pH (5.5 and 6.5), a burst release (80% and 55%) was observed in 3 h. NPSL showed a similar slow drug release profile at pH 7.4 with faster drug release than PSL at pH 6.5, but slower drug release than PSL at pH 5.5.

### *In vitro* cytotoxicity

Following an 18 h exposure of EMT6-*nfsB* cells to liposomal formulations, SRB assay demonstrated significantly higher cytotoxicity of PSL over NPSL and free drug, for which the IC_50_ was 31.06 ± 0.13 µM, 4.71 ± 0.11 µM and 0.22 ±  < 0.01 µM, respectively (mean ± SEM), respectively (Fig. 1D).

Blank liposomes (either containing calcium acetate or PBS) at the corresponding concentrations did not affect proliferation of EMT6-*nfsB* cells following an 18 h exposure.

### Safety, tumor accumulation and anti-tumor efficacy

Following intravenous injection of either liposomal formulation at a high dose 1 mmol/kg (512 mg/kg) of SN256860 in BALB/c or CD-1 mice, no clinical signs or mortality was observed in any animals over the 2 week observation period. However, mice dosed with the free drug solution show a lower body weight in the first week (approximately 1 g) and low movement before recovering in the second week.

**Fig. 2:**
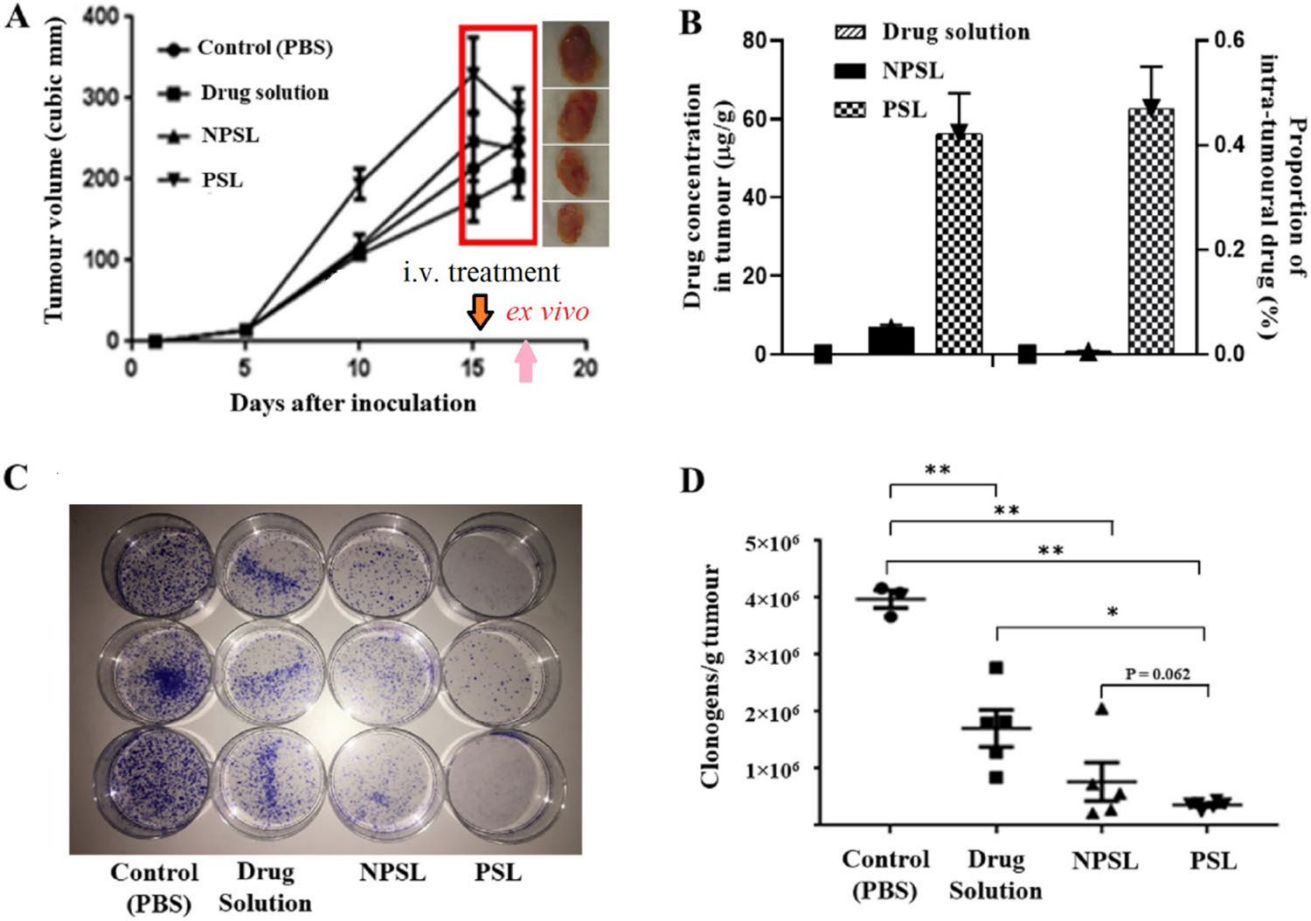
The anti-tumor and tumor targeting abilities in CD-1 nude mice following a single intravenous treatment with SN25860 formulations: drug solution, NPSL and PSL with PBS as control. **A** Change of tumor volume over 2 weeks after inoculation (*n *= 6–7). Box highlights the change in 48 h following a single treatment with SN25860 formulations at a dose of 1 mmol/kg (512 mg/kg) compared with control. **B** Intra-tumoral SN25860 concentrations (*n* = 3), and proportion of intra-tumoral drug to the administered dose after 48 h (ND: not detectable, below 15 ng/g). **C** Representative pictures of clonogenic plates showing median tumor cell colonies grown from mice following different treatment. **D** Anti-tumor activity of SN25860 formulations against subcutaneous EMT6-*nfsB* tumors by *ex vivo* clonogenic assay 48 h after a single IV dose (*n* = 3 for control group; *n *= 6–7 for drug treated groups). Line = geometric mean; bars = SEM. Panel D shows p-values from one way ANOVA with Tukey’s post hoc test for multiple comparison

The tumor targeting abilities of PSL and NPSL were determined by analyzing the amount of SN25860 in EMT6-*nfsB* breast cancer tumors by HPLC 48 h after a single IV injection of 1 mmol/kg in CD-1 mice, once tumor size reached approximately 200 mm^3^ (Fig. 2A). The separate pilot study (*n* = 3) demonstrated that the concentration of the free drug solution group was not detectable in tumor tissues (i.e. below 15 ng/g), nor in other organs including liver (Fig. 2B). Notably, the intra-tumoral drug concentrations in mice treated with PSL was 56.3 ± 10.3 µg/g, ninefold higher than for NPSL treated (6.76 ± 0.76 µg/g) (*p* = 0.001), which is equivalent to 0.47% and 0.06% of the total injected dose. For all animals, drug concentrations in heart, spleen and kidneys were under detection limit.

The anti-tumor efficacy of SN25860 formulations in EMT6-*nfsB* breast cancer CD-1 mice was tested by analyzing tumor volume and clonogenicity in *ex vivo* assays (Fig. 2C-D). Fifteen days after inoculation, tumor size reached approximately 200 mm^3^ and mice received a single IV injection of 1 mmol/kg SN25860, with PBS as control. Interestingly, 48 h after dosing, tumor sizes decreased for PSL and NPSL treated animals while tumors in the PBS and free drug solution groups continued to increase. However, there was no statistically significant difference (*p* > 0.05) compared with the untreated in the tumor reduction ratio.

For *ex vivo* clonogenic assay, tumors were collected 48 h after dosing and cells were isolated to plate as colonies. The number of colonies grown *ex vivo* for all plates was manually counted. An image representative of plates in the different treatment groups, control (PBS), drug solution, PSL-SN25860 and NPSL-SN25860, is shown in Fig. 2C. Notably, the PSL-SN25860 treatment (*n* = 7) provided greater clonogenic cell kill by 91% compared with the PBS group, with few colonies formed in the plates in all animals (Fig. 2D). The colony count in tumors from PSL-treated mice was 4.7 times lower than in tumors from mice treated with the free drug solution (42.7% proliferating cells remaining) (*p* < 0.01), and 2.1 times lower than tumors from NPSL-treated mice (19.4% proliferating cells remaining), although the difference between the PSL and NPSL groups was not statistically significant (*p* > 0.05). Notably, there was smaller individual variation in the PSL group (*n* = 7), compared to the NPSL group (*n* = 6) and the drug solution group (*n* = 3).

### Visualizing the influence of calcium in liposomal core by live cell imaging

**Fig. 3:**
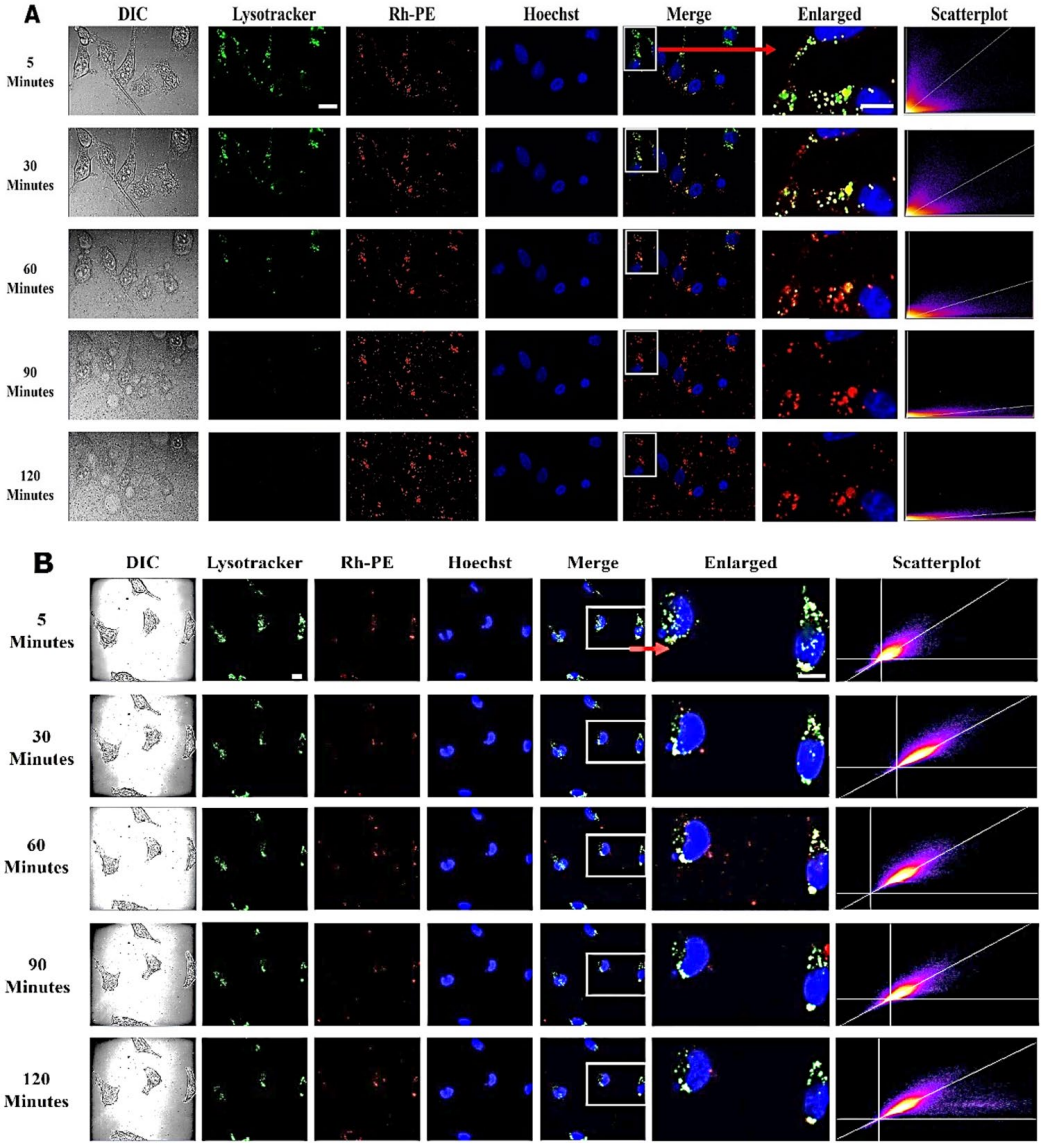

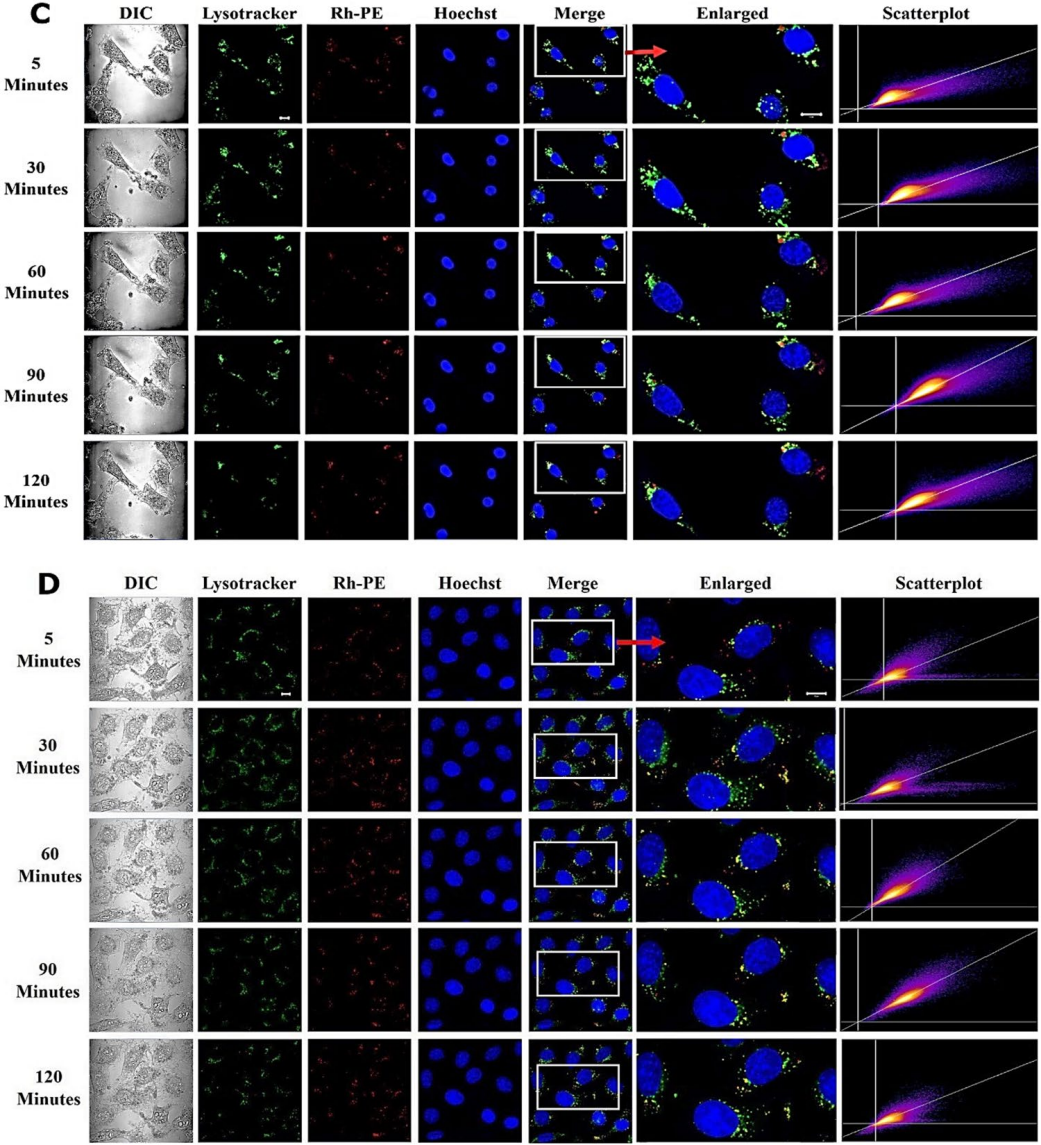
Live cell imaging of EMT6-*nfsB* cells co-existed with PSL (A and B) or NPSL (**C** and **D**) containing 500 mM calcium acetate (**A** and **C**) or PBS of pH 7.4 (**B** and **D**), DIC (grey images) show cell morphology. Rh-PE (red counterstain) was used to label liposomes. Hoechst 33,342 (blue) was used as the nuclear stain. LysoTracker (green counterstain) indicates late endo/lysosomes. The scatterplots were generated by correlation of liposome (Rh-PE) with LysoTracker channel. It was clear that at the dose studied, PSL-Ca treatment caused depletion of endo/lysosomes, which became evident from 60 min onward. Scale bars = 20 µm

To reveal the calcium effect on endosome escape of liposomes, EMT6-*nfsB* cells were treated with Rh-PE labelled PSL and NPSL both containing calcium acetate (500 mM), denoted as PSL-Ca and NPSL-Ca respectively, and imaged with live cell confocal microscopy. Untreated cells containing only LysoTracker served as a control and cells treated with liposomes containing PBS (0.1 M, pH 7.4) i.e. PSL-PBS and NPSL-PBS served as references. These liposomes were almost identical in size but both calcium-containing liposomes were slightly positively charged (Table 1). The endo/lysosomes in EMT6-*nfsB* cells were found to be approximately 1 µm in diameter and well distributed in the cytoplasm (Fig. 3).

PSL-Ca demonstrated rapid uptake by the cells as red fluorescence (Rh-PE) was observed as early as within 5 min (Fig. 3A). Yellow fluorescent spots indicate co-localization of the PSL (Rh-PE, red) with endo/lysosomes (LysoTracker, green), due to the transient entrapment of PSL-Ca in endosomes. After 30 min, the intensity of yellow fluorescence started to decrease leaving behind a bright red Rh-PE fluorescence. Remarkably, the intensity of LysoTracker dramatically decreased from about 60 min and became invisible by 90 min. Meanwhile, Rh-PE fluorescence congruent to PSL-Ca lipid membrane was observed in the cytosol with no change over 2 h. This suggests the rupture of late endosomal (pH < 6) and lysosomal (pH < 5.5) membrane. This is verified with the scatterplots of red (liposome) and green (endosome) channels plotted in which distribution of pixels between the axes demonstrate the correlation of the two channels with the green channels decreasing over time.

In contrast, cells treated with PSL-PBS exhibited a constant strong LysoTracker intensity over time, indicating the presence of undamaged endosomes or lysosomes (Fig. 3B). All scatterplots displayed a bright and grouped pixels indicating strong co-localization all the time as indicated by the yellow pixels. Likewise, cells treated with NPSL-Ca and NPSL-PBS were also observed to have stronger signals for co-localization (yellow) and LysoTracker (green) over time (Figs. 3C-D). These results provided a strong evidence of endosome entrapment of PSL-PBS, NPSL-Ca, and NPSL-PBS.

**Fig. 4:**
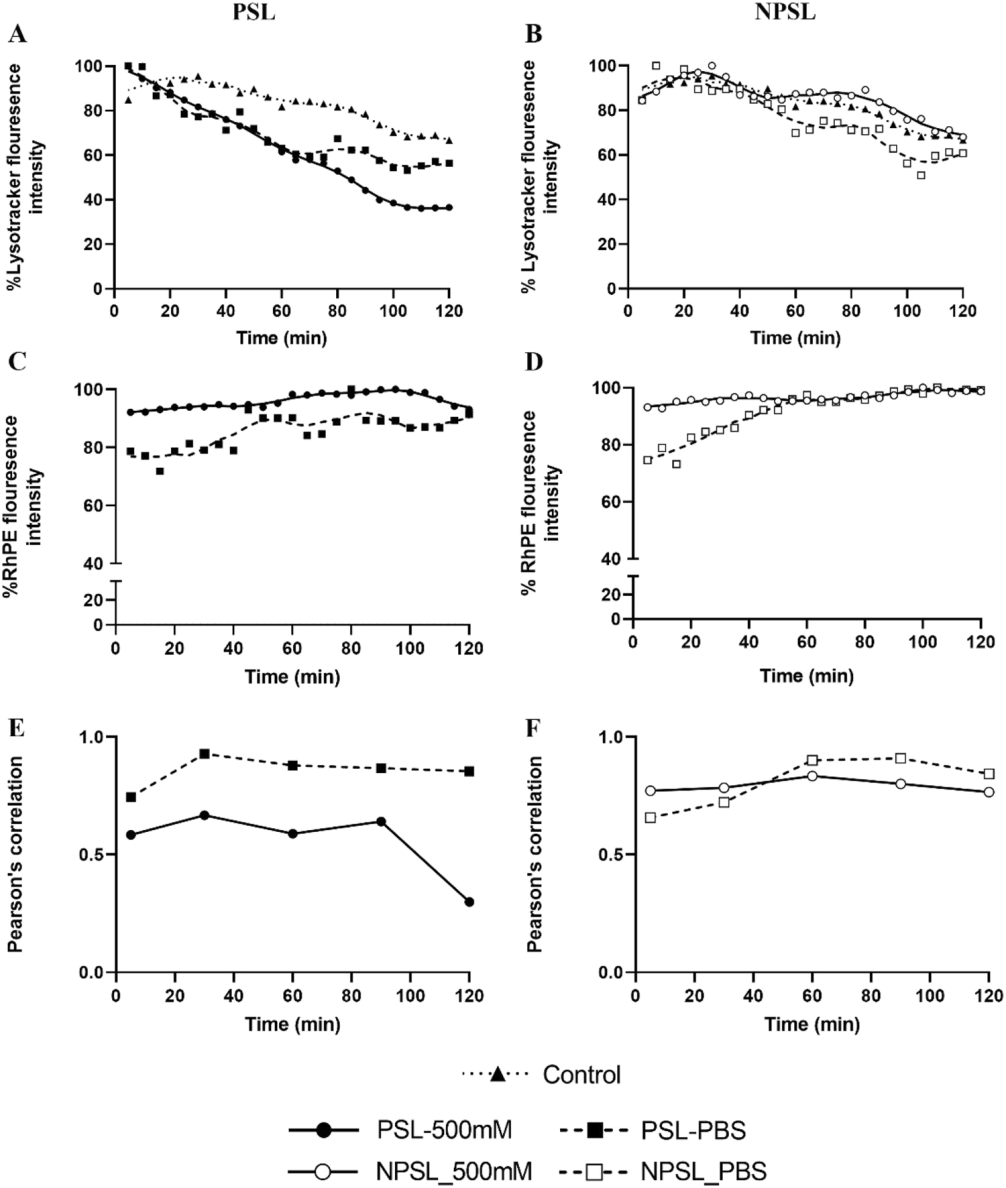
Fluorescence intensities (FI) of Rh-PE and LysoTracker in EMT6-*nfsB* cells co-existed with various liposomes. **A-D**) normalized FI to the respective peak value; **E** and **F**) Pearson’s correlation for co-localization of liposomes and endo/lysosomes

The fluorescence intensities (FI) of LysoTracker and Rh-PE in the liposome-treated cells from the above experiment were quantified and presented in Fig. 4. Overall, a trend of reduction in LysoTracker FI was observed with untreated and treated cells over time. Corresponding to the confocal images, cells treated with PSL-Ca were shown to lose LysoTracker fluorescence linearly over the 120 min in contrast to the untreated, and other formulation-treated cells (Figs. 4A and B). High Rh-PE FI starting from 5 min was observed for cells treated with PSL-Ca as well as NPSL-Ca and both maintained at the same high level over the 2 h. The corresponding liposomes without calcium started with a relatively lower Rh-PE signal with gradual trend of increase with time and eventually reached the same levels as the calcium-containing liposomes (Figs. 4C and D).

As indicated from Pearson’s correlation coefficients (Fig. 4F and E**)**, co-localization of liposomes (Rh-PE) and endo/lysosomes (LysoTracker) for cells treated with PSL-Ca was relatively low compared to the other formulations and stayed constant for 90 min. Again, this may indicate the rupture of endo/lysosomes and/or leakage of endo/lysosomal content to the cytosol for PSL-Ca. In contrast, for cells treated with the other three types of liposomes, the co-localization was higher compared to PSL-Ca, indicating presence of liposomes within the endosomes (endosomal entrapment).

**Fig. 5:**
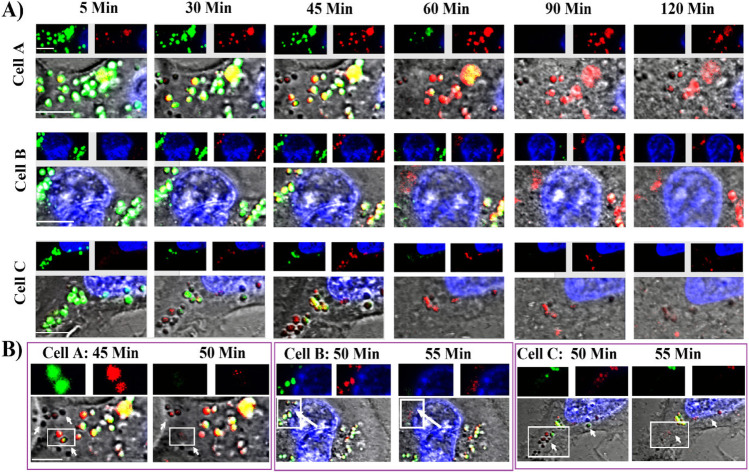
The representative images showing PSL containing 500 mM calcium acetate induces rupture of endo/lysosomes from three different representative cells. (**A**) The endo/lysosomes of cells were pre-stained with LysoTracker (green counterstain) and treated with PSL dyed with Rh-PE (red counterstain) and the interaction was monitored by live cell imaging. Pictures of cells at 5 min show few endo/lysosomes contain liposomes (red signal). (**B**) At around 45–55 min, the endo/lysosomes lose fluorescence signals and DIC images (bottom images) indicate loss of endo/lysosome morphology. Representative signals from individual fluorescence channels of the boxed area are shown in upper panels. The endo/lysosomes were observed to have strong presence of liposomes (red fluorescence) just before the rupture

To further confirm the endosomal disruption effect of PSL-CA, the individual fluorescence signals and DIC images showing endosome morphology of cells treated with PSL-Ca at different time points were carefully examined for any loss in fluorescence signals and/or loss in morphology (Fig. 5A). A strong LysoTracker fluorescence was observed at 5 min with some spots disappeared as early as 30 min (Cell A). The highest Rh-PE FI and bright yellow spots was observed at 45 min or 50 min, indicating liposomal uptake and increased co-localization. Corresponding DIC images also confirm the simultaneous loss of morphology of endosomes within just in 5 min (Fig. 5B).

**Fig. 6:**
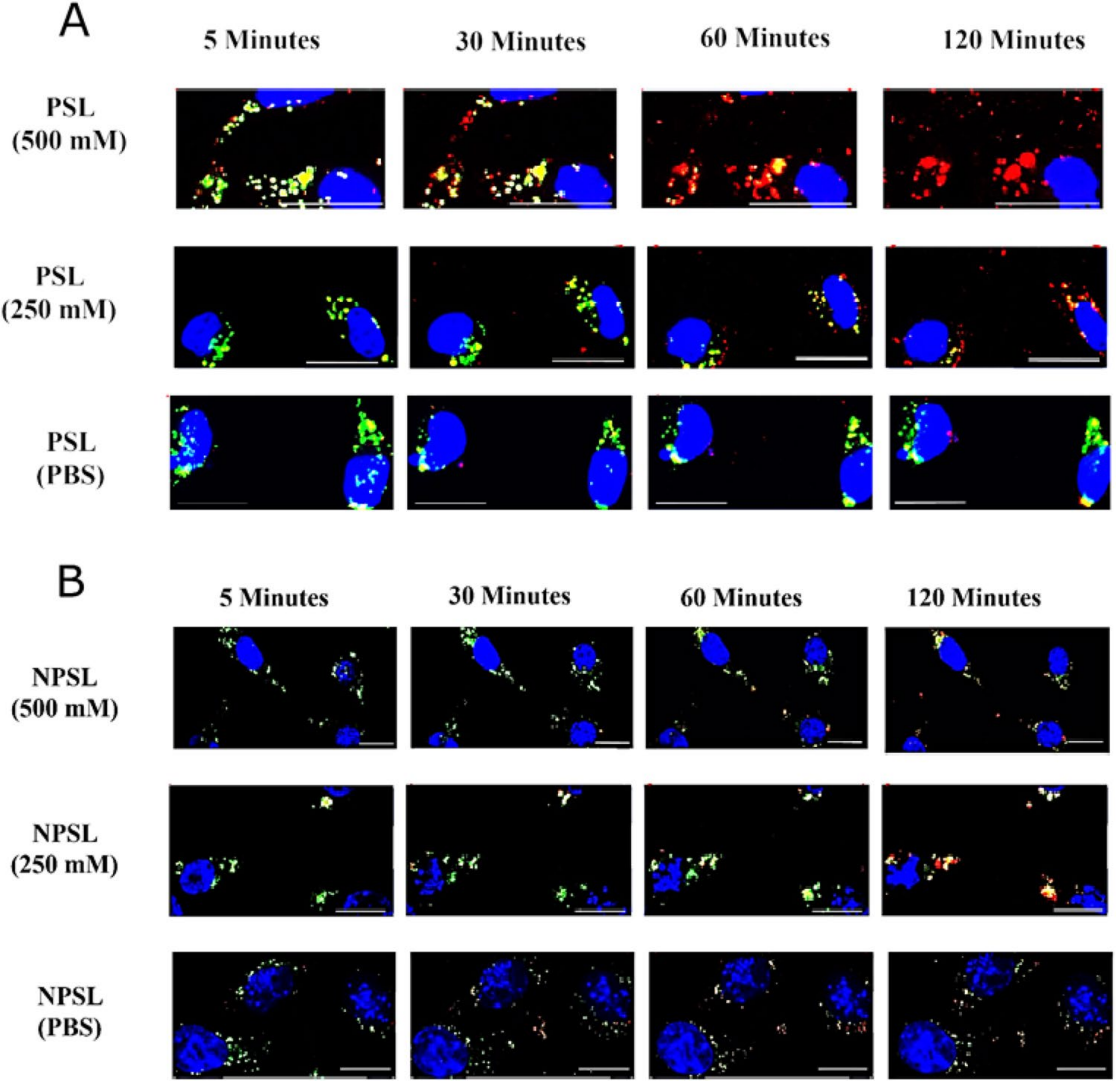
Images of EMT6-nfsB cells co-existed with PSL (**A**) or NPSL (**B**) containing a 250 mM or 500 mM calcium acetate solution or PBS (0.1 M, pH 7.4). Rh-PE (red counterstain) was used to label liposomes. Hoechst 33,342 (blue) was used as the nuclear stain. LysoTracker (green) indicates late endo/lysosomes. PSL-CA treatment caused depletion of late endo/lysosomes, which became evident from 60 min or 120 min depending on the concentration of calcium acetate. Scale bars = 20 µm

In another experiment, PSL and NPSL were loaded with calcium acetate (250 mM or 500 mM) or PBS (0.1 M pH 7.4) before exposed to EMT6-*nfsB* cells. Figure 6, only PSL containing calcium acetate caused rupture of endo/lysosomes, which became evident from 60 min when intraliposomal concentration of calcium acetate was 500 mM. This is delayed as calcium reduced to 250 mM.

## Discussion

A great interest and pursuit of successful nanotechnologies continues to realize the full potential of nanomedicines [2, 3]. Too slow drug release in cancer cells is one of the major reasons for nanomedicine failure [40]. Overcoming the ‘endosomal-entrapment’ may have significant impact on efficacy of the current nanomedicines [41] and create new generation nanotherapeutics [42, 43]. In this study, with a translational view in mind, we developed compositionally facile liposomes for delivery of SN256860 endowed with multiple endosomal-escaping mechanisms while also addressing some other major formulation challenges—inferior drug loading and poor cellular uptake [10, 44]. As PEGylation reduces cellular uptake by cancer cells [10], the PEGylation degree was tailored to 3% mol (rather than the commonly used 5%) to balance the cellular uptake and long-circulation [14].

### Calcium acetate enabled high drug loading into liposomes

To increase the intracellular delivery efficiency a high drug content is crucial[5] given the cellular uptake could be saturable. Remote loading drug into PSL is technically challenging due to the small workable pH window in addition to the limited drug solubility [19]. In this study a high DL of 31.1% (w/w) for PSL and 33.2% for NPSL as well as high EE was achieved (Table 1) with the aid of a combined strategy **(**Fig. 1A). This included obtaining a highly concentrated SN25860 (p*K*_a_ 4.16) solution for drug loading (4.5 mg/ml) with multiple approaches: HP-ß-CD, co-solvency and pH-control. The HP-ß-CD stabilized the super/saturated drug solution from precipitation [45], and maintained a rich drug reservoir for influx [46]. The pH-control maintained a stable pH-gradient across the liposome membrane. The high EE was also attributed to a sufficient concentration of calcium acetate (500 mM, pH 9) to establish a driving force for drug-influx (Fig. 1A). Furthermore, Ca^2+^ may sequester a large amount of drug inside liposomes by forming a less-soluble complex [47], evidenced by the ‘bundles’ of drug precipitates in cryo-TEM images (Fig. 1B). No bundles were observed in the SN25860 liposomes when DL was 7% [19].

*In vitro* release studies demonstrated the pH-responsive release of the amphiphilic weak acid SN28560 from PSL (Fig. 1C). At low pH, the charged head groups of DOPE are protonated, promoting the PSL structure to collapse^21^, thus, in the endosomes (pH 5.0–6.5) PSL released their payload rapidly. Compared with the liposomes with 7% SN28560 [19], increasing DL led to better drug retention in both PSL and NPSL at pH 7.4 (30% entrapped after 24 h), possibly due to the precipitation as suggested in TEM (Fig. 1B). Drug retention in liposomes during circulation in blood is essential for tumor-targeting by exploiting the long circulation time of nanoparticles [38]. The NPSL containing DPPC also presented pH-responsiveness, as found before with low DL of SN28560 in liposomes [19]. However, release of hydrophilic compounds carboxyfluorescein [15] or calcein [16] in the aqueous cores of DPPC liposomes was not pH-dependent as DPPC bilayer is not pH-responsive. Thus, protonation of the ionized drug species, and dissociation of SN28560-Ca^2+^ complex due to H^+^ penetration inside the liposomes might have contributed the rapid release at low pH.

### Cytotoxicity and the drug loading effect

Following an exposure to EMT6-*nfsB* breast cancer cells for 18 h, PSL-SN25860 demonstrated a 21.4-fold and 141.0-fold decrease in IC_50_ compared to NPSL-SN25860 and the free drug, respectively (Fig. 1D). Both liposomes facilitated the intracellular delivery SN25860 (a weak acid with *p*K_a_ 4.16) which is otherwise hindered by its ionization in extracellular media (pH 7.4). The significantly higher cytotoxicity of PSL than NPSL was attributed to the fusogenic property and endosomal escape abilities of PSL. Interestingly, compared with the PSL and NPSL with DL of 7% (IC_50_ 1.28 ± 0.8 μM and 27.1 ± 4.2 μM) [19], increasing DL by 4.4-fold resulted in a 5.8-fold increase in cytotoxicity for both liposomes. Given liposome uptake into cells is considered to be via energy-dependent endocytosis [48], a higher DL leads to a greater amount of drug to be taken up by cells using the same amount of energy for endocytosis [49].

### Safety, tumor accumulation and anti-tumor efficacy

Following a treatment with each formulation at a high dose (512 mg/kg) of SN256860, no mortality was observed in any animals in two weeks, suggesting both the drug SN256860 designed as a bioreductive prodrug utilizing tumor hypoxia [26] and liposomes are well tolerated at this dose level. This is further conformed in the efficacy study where none of the animals showed signs of discomfort after treatment.

In the polit study (*n* = 3), intra-tumoral drug concentration 48 h post drug injection was undetectable (below 15 ng/g) for the free drug treated animals but was at least 450-fold higher (6.76 ± 0.76 µg/g) for the NPSL group and at least 3700-fold higher for PSL group (Fig. 2), verifying the superior tumor-targeting ability of liposomes. The intra-tumoral drug corresponds to 0.06% and 0.56% of the injected dose (ID) for NPSL and PSL, respectively. The current understanding based on existing data is that maximal 0.76% ID (median) or 2.24% ID (mean) of nanoparticles can be found in tumors at 24 h post-injection and declines over time [50, 51]. This measurement reflected the total extracellular and intracellular drug concentrations. The tumor accumulation is highly dependent on particle size, composition and surface properties (charge, PEGylation, ligand) of the nanomedicines, and even tumor models. A threshold of the particle number exists to saturate the uptake by Kupffer cells for liver clearance, beyond which the delivery efficiency would be increased as dose is increased [52]. Thus, the same number of nanoparticles with high DL would lead to high drug concentration in tumors. Given that particle size, zeta potential and long-circulation were almost identical for PSL and NPSL (Table 1), the same amount of tumor accumulation was expected. This 9.3-fold discrepancy is most likely attributed to the endosome escape of PSL which evaded lysosomal drug degradation. Furthermore, the fusogenic properties of DOPE in PSL could have facilitated cellular uptake after extravasation from blood vessels. Figure 1D shows that with the same extend of drug exposure to cells, PSL produced a 21.4-fold potency than NPSL, attributing to its efficient cytoplasmic release.

In the tumor-bearing mice, a single treatment with PSL-SN25860 appeared to reduce tumor size within 48 h (although p > 0.05), to a greater extent than NPSL-SN25860, while tumors in PBS and free drug solution treated-mice continued to grow (Fig. 2A). *Ex vivo* clonogenic assay showed a high number of colonies in free drug, even NPSL-SN25860 groups. This is due to the heterogenicity of tumors which contain sensitive cells and subsets of drug-resistant cells such as cancer stem cells (CSC). Of note, PSL-SN25860 almost ablated all clonogenic tumor cells in all seven treated animals (Fig. 2C and D). Even with limited tumor penetration due to the particle size of liposomes [53] regionally high concentrations of the prodrug and possibly its activated metabolites would allow diffusion to the deeper tissue exerting cytotoxic effects. Therefore, tumor shrinkage may be expected to become more significant after 48 h for the PSL-SN25860 group.

### Mechanism of endosomal escape and calcium effect

The above results demonstrated that the PSL may have significantly better endosome escape ability empowered by calcium inside the liposomes. Endosomal escape has been long recognized as an important strategy to ensure cytosolic delivery of therapeutics, and several strategies can be employed including pH-responsiveness [12, 13] and “proton sponge effect” pathway [54]. However, to date, there is no substantial evidence in literature revealing the endosomal escape process. Therefore, in this study, dynamic interaction of the EMT6-*nfsB* cells with PSL and NPSL containing calcium acetate (500 mM) visualized by live-cell confocal microscopy, provided first visual evidence to a proton sponge effect inducing endo-lysosomal rupture.

### Calcium promoted liposomal internalization irrespective of pH-sensitivity

As expected, there was a superior cellular uptake of PSL over NPSL by EMT6-*nfsB* cells, (Figs. 3, 4, 5 and 6). Moreover, as suggested by the Rh-PE signals, both PSL-Ca and NPSL-Ca internalized to their highest level instantly (in 5 min) while PBS-liposomes took 1 h to reach the peak level. Ca^2+^ (but not other bivalent cations like Zn^2+^) has been shown to bridge clathrin on cell membranes with Ca^2+^-siRNA nanocomplexes and promote clathrin-mediated endocytosis  [29], a major cell-entry pathway for liposomes [19]. Ca^2+^ was likely to leach out from the liposomal core, supported by the slightly increased zeta potential of PSL-Ca and NPSL-Ca (by 2–3 mV) compared to corresponding PBS liposomes (Table 1). This increase in positive charge also favors cellular uptake via electro-static interactions with the negatively charged cancer cells [55].

### The mechanism for endosomal escape mediated by calcium

All other types of liposomes showed stronger signals for co-localization and LysoTracker over time, suggesting endosome entrapment [22]. While Rh-PE signal was stable (or increase over time due to continued cellular uptake) there was a significant loss of LysoTracker fluorescence overtime for PSL-Ca (500 mM) treated cells, at a higher rate than PSL-PBS (Figs. 4A and B). However, the co-localization in PSL-Ca (500 mM) treated cells dramatically dropped at 1 h. The gradual loss of LysoTracker signal (pH-dependent) could suggest either direct neutralization of the endo/lysosomal content by released calcium acetate (pH ~ 8) or PBS (pH 7.4) from PSL. For PSL-Ca, it could also be leakage of LysoTracker due to endo/lysosomal swelling, or even rupture via the proton sponge effect. Indeed, detailed imaging analysis suggested disruption of some endo/lysosomes after 45 min upon liposome-cell exposure of PSL-Ca (500 mM) (Fig. 5). This rupture event was not observed in cells treated with PSL-PBS or NPSL-Ca. Reducing the concentration of entrapped Ca^2+^ caused delay of this rupture by 1 h (Fig. 6A).

From the above analysis, it can be hypothesized that endosomal escape of endocytosed PSL-Ca was accomplished by two steps sequentially: 1) destabilization of PSL in the acidic endo-lysosomal lumen, allowing release of payload including Ca^2+^; and 2) free Ca^2+^ induced endosomal swelling, or even rupture via the ‘proton sponge effect’^29,30^. This explains the lack of endosomal rupture by NPSL-Ca or PSL-PBS where no free Ca^2+^ ions are available inside endosomes. Alternatively, the fusogenic properties of DOPE may allow some PSL fuse with endosomal membrane, releasing the payload to cytoplasm.

It is worth pointing out that in this live cell imaging study, the liposomal core had a pH of 9 which should have reduced the pH-sensitivity and retarded their endosomal escape. After drug is loaded, the intraliposomal pH is expected to drop allowing the pH-sensitivity of liposomes to be restored, thus in theory PSL-SN25860 should outperform PSL-Ca for endosome escape *in vitro* and *in vivo*. It was also observed that the florescent phospholipid, Rh-PE was preserved in the cells, with no significant change in its overall FI over the 2 h even if liposomes were entrapped, fuse or rupture with endo/lysosomes. In contrast, LysoTracker intensity decreased over time in all the cells including the untreated cells. This may be explained by endosome leakiness [56], or secretion via extracellular vesicles (exosomes) [57].

## Conclusions

In this study, calcium acetate has been demonstrated to possess multifaceted functions in promoting drug loading of amphiphilic weak acids into PSL, cellular uptake, and endosomal escape, and ensured efficient cytoplasmic delivery of SN28560. A high DL (> 30%) and EE (> 95%) of a weak acid SN28560 was achieved for PSL and NPSL. Both liposomes demonstrated increased blood circulation compared to free drug solution, with less liver accumulation. *In vitro* cytotoxicity and tumor accumulation of PSL-SN25860 were significantly greater compared to NPSL or free drug which led to a significantly greater anti-tumor effect. Further, live cell imaging provided evidence that Ca^2+^ in PSL, but not NPSL, induced the endosomal disruption in EMT6-*nfsB* cells, as proposed by the ‘proton-sponge effect’, and facilitated a rapid drug release to cytosol.

Taken together, the PSL containing calcium possess superior capabilities for tumor targeting followed by endosomal escape. This could shed light in designing nanomedicine platforms for delivery of a wider range of therapeutics that necessitate cytoplasmic delivery including DNA toxins as well as for nucleic acids (DNA, siRNA and mRNA).
